# Lentiviral-Mediated RNAi Knockdown of Cbfa1 Gene Inhibits Endochondral Ossification of Antler Stem Cells in Micromass Culture

**DOI:** 10.1371/journal.pone.0047367

**Published:** 2012-10-09

**Authors:** Hongmei Sun, Fuhe Yang, Wenhui Chu, Haiping Zhao, Chris McMahon, Chunyi Li

**Affiliations:** 1 Institute of Special Wild Economic Animals and Plants, Chinese Academy of Agricultural Sciences, Changchun, Jilin, P. R. China; 2 AgResearch, Invermay Agricultural Centre, Mosgiel, New Zealand; 3 State Key Laboratory for Molecular Biology of Special Economic Animals, Jilin, P. R. China; University of Pittsburgh, United States of America

## Abstract

Articular cartilage (AC) lacks ability to repair defects due to its avascular nature as healing process relies on cells being brought in by blood vessels. Multiple approaches have been taken to facilitate cartilage repair in clinics, to date there is no effective treatment available that can restores the AC lesion to a normally functioning level over extended periods. In this regard, antler cartilage is unique in being richly vascularised and hence can effectively repair and regenerate. Interestingly, antler stem cells, from which the vascularised cartilage is derived, can form avascular cartilage when taken away from their original niche, suggesting that the vascular or avascular state of antler cartilage is controlled by extrinsic factors. Understanding the mechanisms underlying this phenotype switch may help us to devise a way to trigger the effective intrinsic repair of AC. However, adoption of antler cartilage model for AC repair requires the demonstration that the cartilage specific signalling pathways also prevail in antler chondrogenesis. To achieve this, in the present study we silenced expression of Cbfa1, a key factor regulatingendochondral ossification, using RNAi, and showed that expression of the downstream genes type I collagen and osteocalcin were suppressed which, in turn, inhibited endochondral ossification process taking place in the antler stem cell-formed nodules. Therefore, we provided further evidence at molecular level that antler could be developed as novel model for the study of AC repair. The eventual identification of the extrinsic factors dictating the phenotype switch between the vascular and avascular state of antler cartilage will open up a new avenue for the cure of osteoarthritis.

## Introduction

Articular cartilage (AC) is a type of truly extraordinary tissue in that it tolerates a tremendous amount of intensive and repetitive physical stress, but manifests a striking inability to heal even the most minor injury [1]. The inability to heal is attributed to two important features of AC: cell immobility and avascularity, the later being more important [2]. It is known that effective tissue repair requires the presence of specific cells to clean up necrotic material and to synthesize new tissue. These cells are either derived from those that have migrated from the wound margin or enter the area by blood vessels. Due to the avascular nature of AC, cells needed for repair cannot be brought in through blood vessels; moreover, the chondrocytes in AC are literally imprisoned in a mesh of collagen and proteoglycan, and are unable to migrate from adjacent healthy cartilage to the site of injury where they are needed for repair. Therefore, strategies devised to repair AC have thus far focused on either facilitating access to an adjacent vascular supply or via physical delivery of new cells capable of promoting chondrogenesis.

To gain access to the vascular system, drilling through subchondral bone arguably the most effective way to expose the AC to blood vessels, and hence to trigger the intrinsic cartilage repair. However, chondrogenesis initiated via this way can only provide an inferior and transient fibrocartilagenous replacement for hyaline cartilage, but not durability of biomechanical function [3]. Multiple approaches for transplantation of cells to the damaged AC have been attempted, and these have focused on the delivery of either native tissue, such as periosteal or perichondreal flaps [4], [5] or synthetic biomaterials, such as collagen scaffolds [6]. However, one characteristic shared by all these reparative processes, besides the inherited problems associated with each approach, is an apparent lack of lateral integration (microfractures or large fissures) of repair or grafted tissue with the host cartilage that normally lead to poor prognosis [2].

Research into the role of growth factors in cartilage homeostasis and repair [7], gene therapy [8] and biomaterial development [9] have significantly improved the outcomes of AC repair. However, there is no current method that has convincingly demonstrated that repair of AC tissue to a normally functioning level over extended periods [10]. Undoubtedly, the development of a therapeutic modality for permanent restoration of injured AC must be based on the sound understanding of the basic biology of this particular tissue. In this regard, animal models have played and would be continuously to play a critical role in identifying the mechanisms of repair and in testing treatment options for restoring the function of AC after injury [11].

Amongst the animal models, deer antlers are unique in that its cartilage can not only fully regenerate, but regenerate at a phenomenal speed (up to 2 cm/day; [12]). This remarkable ability of antler cartilage can only be attributed to its distinctively-characteristic structure, i.e. cartilaginous tissue infiltrated with an extensive vascular network [13], [14]. Antlers are organs of bone and regenerated from the periosteal cells of a pedicle [15], the permanent bony protuberance from which an antler drops off and regenerate [16], [17]. Pedicle periosteal cells express key embryonic stem cell markers (such as Oct4, Nanog and Sox2) and can be induced to differentiate into multiple cell lineages (such as chondrocytes, adipocytes, myotubes and neuronal cells), therefore, these cells are called antler stem cells ([18], [19]. Cartilage of both somatic and antlersis formed through endochondral ossification (EO), butthere is a key difference between these two types of EOs. In the somatic EO, mesenchymal cells firstly form cartilage (i.e. the avascular tissue) and then vascular system invades into the tissue, which leads to the eventual replacement by bone. In contrast, antler mesenchymal cells form firstly vascularised preosseous tissue that is then replaced by bone without being subjected to the process of vascular invasion [13]. Because of the vascular nature, whether antler preosseous tissue was true cartilage had been the bone of contention over the last century and half [20]. After confirming the cartilaginous nature of antler preosseous tissue ultrastructurally [21] and histochemically [22], Banks and Newbrey [20] concluded that this tissue is true cartilage, and termed the process of antler cartilage formation a type of “modified” EO. Further, those authors inferred that the vascular status of antler cartilage may be the reflection of the high metabolic demands for the fast growth.

When carrying out the experiments to characterise antler stem cells, Li et al found that these cells could form avascular cartilage if they were removed from the original site and cultivated in a less favourable milieu for growth, such as in a diffusion chamber in vivo [23], in a nude mouse [24] or in vitro in a micromass culture [25]. These results clearly demonstrate that formation of vascular or avascular cartilaginous tissue by antler stem cells can be dictated by altering the environment in which these cells reside. Understanding the mechanism underlying this phenotype switch could open a completely new avenue for AC repair. However, we recognised a need to further evaluate whether antler chondrogenesis was mediated by the same molecular signalling pathway that prevails in the somatic EO before antler cartilage model could be applied in the investigation of AC repair.

One of the key molecules in the signalling pathway of EO is Cbfa 1 (core binding factor a1), also known as RUNX2 [26]. Cbfa 1 is a transcriptional factor and mice with targeted Cbfa1 gene mutation showed a complete lack of bone formation, suggesting that Cbfa 1 is indispensible in osteoblast differentiation [27]. Further studies showed that Cbfa 1 positively regulates chondrocyte hypertrophy [28], extracellular matrix mineralisation, and expression of bone matrix genes including osteopontin, bone sialoprotein (BSP), osteocalcin and type I collagen [29]. Therefore, if silencing Cbfa1 gene in antler stem cells would result in suppression of Cbfa1 downstream gene expression, and impediment of differentiation of cartilage and bone tissue of antlers, we would have provided further evidence that antler EO is also realised through the well-established signalling pathway although its cartilage is vascularised.

RNA interference (RNAi) technology is now widely used to study gene function, as it allows expression of a single gene transcript and protein product to be efficiently and specifically reduced or knocked down at the mRNA level [30]. This technique provides the opportunity to study the aforementioned effects of Cbfa1 in antler modified EO.

The aims of the present study were to use RNAi technique to silence the Cbfa 1 gene expression in antler stem cells in a micromass culture in vitro to determine whether, 1) expression of the down-stream genes, osteocalcin and type 1 collagen, would be down-regulated in antler stem cell-derived cartilage; 2) differentiation of the antler stem cells towards cartilage and bone would be impeded. The results should point out whether antler EO was comparable to the classic counterpart at molecular level, and thus provide evidence whether the mechanism underlying phenotypic switch in antler cartilage case can be applied to AC repair.

## Materials and Methods

### Pedicle periosteum sampling and antler stem cell culture

Periosteum was collected from both sides of pedicles of a two year old male sika deer immediately after slaughtering in Zuojia Deer Slaughtering Plant using aseptic technique as described previously [31]. Briefly, after shaving and sterilizing the enveloping skin each pedicle was cut open to expose the periosteum, which was then divided into strips around 5 mm in width along the longitudinal axis of the pedicle using a scalpel. The periosteal strips were peeled off from the pedicle bone using a pair of rat-toothed forceps and then placed into a 50 ml centrifuge tube containing 20 ml DMEM medium plus 500 U/ml penicillin and 500 g/ml streptomycin (Invitrogen, USA).

For antler stem cell culture (also refer to [32]), the periosteal strips were washed three times using the same DMEM medium before being transferred to a 10 cm Petri-dish, where they were cut into small pieces (around 1 mm^2^/piece) using a pair of scalpels. These pieces were digested in the digesting medium (DMEM +150 units/ml collagenase; Invitrogen, USA) at 37°C for 1–1.5 hrto release antler stem cells and then the digest was centrifuged at 1500 rpm. The pellet was resuspended in 10 ml culture medium (DMEM +10% FBS +100 U/ml penicillin +100 µg/ml streptomycin) and transferred into a 75 ml flask (Nunc, Danmark) containing 20 ml culture medium. The antler stem cells were trypsinized upon reaching confluence, and reseeded in T75 culture flasks at 1×10^5^ cells/ml. The cells were detached again when reaching around 85%–90% confluence, transferred to frozen medium (FBS +10% DMSO) at 1×10^6^ cells/ml, and then stored in liquid nitrogen. When needed, the antler stem cells were retrieved from the storage and seeded in T75 flasks containing 20 ml culture medium.

### Micromass culture

Micromass culture was carried out following the method reported elsewhere [33]. Briefly, antler stem cells were cultured in T75 flasks, trypsinized when reached 85–90% confluence, and resuspended in the chondrogenic medium (DMEM +10 ng/ml TGF-β1 +10^−7^ M dexamethasone +50 µg/ml ascorbate-2-phosphate) to a concentration at 1×10^8^ cells/ml. Aliquot of 100 µl of cell suspension was seeded in the centre of each well of a 6-wellplate (Nunc, Danmark) and incubated for 4 hr at 37°C to facilitate the adherence of the cells. Thereafter, 2 ml chondrogenic medium were gently added into each well around the forming cell aggregate. The medium was subsequently replaced every two to three days. The culture was observed daily by phase-contrast light microscopy. The resultant cell nodules were harvested 3–4 weeks after initial seeding.

### Histology and immunohistochemistry (IHC)

For histology, each antler stem cell-nodule was fixed in 10% buffered formalin and embedded in paraffin wax, and sections were cut at 5 µm thick intervals. The nodule sections were stained using haemotoxylin and eosin (for general morphology), and counterstained using alcian blue (for the detection of cartilage matrix) or alizarin red (for the detection of mineralization), and photographed using a light microscope (Leica, Germany).

For IHC, the antler stem cell-nodule sections from the above were collected onto Poly-L-Lysine coated slides. Following rehydration, the sections were incubated with 3% H_2_O_2_ at room temperature for 5–10 min to quench the endogenous hydrogen peroxidise; boiled in 10 mM citrate buffer (pH 6) for 15 min to facilitate retrieving the antigens; incubated with 10% goat serum at room temperature for 10 min to block non-specific bindings; and incubated with the primary antibodies (monoclonal anti-osteocalcin at 1∶500 dilution, Abcam; monoclonal anti-cbfa1 at 1∶100 dilution, Abcam, Hong Kong) at 4°C overnight. The secondary antibody (goat anti-mouse at 1∶200dilution, Abcam, Hong Kong) was then applied to the sections for 30 min at room temperature, then the complex was developed with DAB reagents (Maixin, Fujian China) to form permanent brown precipitate. The sections were examined and photographed using a light microscope (Leica, Germany).

### Deer Cbfa1 gene cloning and sequencing

Total RNA was extracted from the sub-confluent antler stem cells using TRIzol Reagent (Invitrogen, USA) according to manufacturer's instruction. Reverse transcription (RT) was performed using a high fidelity Primescript RT-PCR kit (TaKaRa, Dalian, China) according to the manufacturer's protocol. The specific primers were listed in Table 1. PCR reactions were carried out under the following conditions. A 5-min denaturation step at 94°C; 35 amplification cycles: 10 sec at 98°C, 5 sec at the 55°C annealing temperature, an elongation step at 72°C for 1 min. The PCR products were purified using QIAEX II Gel Extraction Kit (QIAGEN, Germany) and cloned into vector pMD-19T (TaKaRa, Dalian, China). Three to five independent clones of each amplicon were isolated and sequenced by using M13 sequencing primers.

**Table 1:** Primers used for RT-PCR, qPCR and 3′RACE.

PCR/gene	Forward	Reverse
RT-PCR/Cbfa1	GAAGAGGCAAGAGTTTCACC	AGGGTCGCCAGACAGATT
qPCR/Cbfa1	GGTCCGCTCTGGCTTTG	GGCATGTCCCTCGGTATGT
qPCR/Col 1	GGGGCAAGACAGTGATCGAA	TGGAAGGAGTTTACAGGAAGCAG
qPCR/β- Actin	GCGTGACATCAAGGAGAAGC	GGAAGGACGGCTGGAAGA
3′RACE/Cbfa1	AGTCACCTCAGGCATGTCCCTCGGTAT	

To provide sufficient sequence resources for more choices when designing siRNAs, 3′cDNA end of deer Cbfa 1 gene was cloned by using the SMART RACE cDNA amplificatin kit (Clontech, USA) according to manufacturer's protocol. Touchdown PCR procedures were performed in a thermal cycler (ABI, USA) under the following conditions. A 5-min denaturation step at 94°C; 5 cycles at 94°C for 30 s, 72°C for 3 min; and 5 cycles at 94°C for 30 s, 70°C for 30s ,72°C for 3 min; and 35 cycles at 94°C for 30 s, 68°C for 30 s , 72°C for 3 min. A 5 µl of PCR product was electrophoresed on 1.2% agarose gels, stained with ethidium bromide and visualised under a UV transilluminator. The PCR products were purified using QIAEX II Gel Extraction Kit and cloned into pMD-19T Vector. Three to five independent clones of each amplicon were isolated and sequenced by using M13 sequencing primers.

### siRNA synthesis

Six siRNA (small interfere RNA) sequences against deer Cbfa1 mRNA were designed and designated as S1-S6 (Table 2) based on the Angela siRNA design rules and the MIT online tool (http://jura.wi.mit.edu/bioc/siRNAext/home.php). The specificity of all the target sequences and the negative control sequence (scrambled sequence) were confirmed by BLAST search. All siRNAs were synthesized by Shanghai Shenggong Inc, and annealed into double strand small hairpin RNAs.

**Table 2:** Interfering sequences of siRNA targeting for Cbfa1.

group	siRNA	loop
S1	cgcgtccccATATCAACTCGCTTTAACA *ttcaagaga* TGTTAAAGCGAGTTATAT tttttggaaat	ttcaagaga
S2	cgcgtccccATCAACTCGCTATCTACAA *ttcaagaga* TTGTAGATAGCGAGTTGAT tttttggaaat	ttcaagaga
S3	cgcgtcccc CTATCACAGAGCTATTAAA*ttcaagaga* TTTAATAGCTCTGTGATAG tttttggaaat	ttcaagaga
S4	cgcgtcccc GAGAGTGCATATATATGTA*ttcaagaga* TACATATATATGCACTCTC tttttggaaat	ttcaagaga
S5	cgcgtcccc CCAGCCACCTTTACTTACA*ttcaagaga* TGTAAGTAAAGGTGGCTGG tttttggaaat	ttcaagaga
S6	cgcgtcccc ATGCCTCTGCTGTTATGAA*ttcaagaga* TTCATAACAGCAGAGGCAT tttttggaaat	ttcaagaga
scrambled shRNA	cgcgtcccc ACCGTCTGTGTATCGTCGC*ttcaagaga* GCGACGATACACAGACGGT tttttggaaat	ttcaagaga

### Construction of lentiviralsiRNA vector

The lentiviral vector system (gift from Prof. George Liu, Beijing University [34]) was used to deliver siRNA into antler stem cells. The system consists of three plasmids pLVTHM, pCMV and pMD2. G. The plasmid pLVTHM contains human H1 promoter that can drive persistent expression of siRNA gene, and EF1 (Elongation Factor1) promoter for GFP expression. Each siRNA sequence was inserted in the region between Cla1 and Mlu1 (Fig. S1A). The plasmid pCMV encodes necessary viral constitutive genes (Fig. S1B) and the plasmid pMD2G includes VSV-G gene that provides the capsid protein for virus packaging (Fig. S1C). The specific lentiviral vector was constructed as follows: each of seven siRNAs (including six target sequences and one scrambled negative control sequence) was inserted into the pLVTHM at the ClaI and MluI restriction enzyme sites using T4 DNA ligase. The resultant DNA (pLVTHM-siRNA), pCMV and pMD2. G were then co-transfected into 293T cells using lipofectmine 2000 reagent (Invitrogen, USA) according to manufacturer's protocol. The supernatants containing lentiviral particles were collected 24h and 48h after transfection respectively, pooled together, and concentrated by centrifugation using the Amicon ultra centrifugal filter devices (Millipore Corporation, USA). The titer was tested according to the series of dilution methods [35]. The aliquots were then stored at −80°C.

### Lentiviral infection

For passage one, antler stem cells from these subcultures were trypsinized and resuspended in the lentiviral-siRNA-vector supernatants (S1–S6, scrambled siRNA) or normal culture medium (an additional control) to a concentration at 10^8^ cells/ml. The titer of each vector supernatant was tested through a series dilution method and found to be around 10^8^ U/ml; and no significant difference was detected amongst these 7 lentiviral-siRNA-vector supernatants. Micromass culture was carried out for these cells using the way described above. The resultant nodules were monitored regularly and successful expression of siRNA in a nodule was determined by the timing and degree of GFP expression under a fluorescent microscope (Leica, Germany).

### RT-qPCR

Total RNA was extracted from each nodule formed either from the lentiviral infected or uninfected antler stem cells using TRIzol reagent (Invitrogen, USA) according to manufacturer's protocol. To determine the expression levels of Cbfa 1 gene and type I collagen gene in each nodule, RT-PCR and RT-qPCR were performed as described in a SYBR Primescript RT-PCR Kit (TaKaRa, Dalian, China). Primers were designed using software Primer Premier 5.0 (Table 1). Primer sequences were screened using a BLAST search to confirm specificity, and the PCR products with the expected size were detected on an agarose gel. The efficiency of each primer set for RT-qPCR was determined to be between 95% and 100%. Briefly, Total RNA (500 ng) of each antler stem cell-derived-nodule was reverse-transcribed to cDNA at 37°C for 15 min. All qPCR was carried out in 96-well-plates using the ABI PRISM7500 qPCR System. The final reaction volume per well was 50 µl containing 100 ngcDNA, 500 nM forward and reverse primers (Table 1), and 26 µl SYBR Green PCR Master Mix. Each reaction was run in triplicates and β-actin gene was used as an internal control. The expression level (ΔCt) of Cbfa 1 or type I collagen gene was calculated out by subtracting the Ct value of β-actin from the Ct value of each of these genes. The relative level of each gene expression was determined by the comparative Ct method (2-ΔΔCt) (Forienza et al, 2012) and analyzed by the SDS software. The control was normalised to 1 and expression data were presented as bar graphs.

### Western blot analysis

Total protein of each nodule was extracted using the total tissue soluble protein isolation kit (GENMED, USA) according to the manufacturer's protocol. The proteins were then resolved and separated on a sodium dodecyl sulfate-10% polyacrylamide gel by electrophoresis, and then transferred to aCellulose Nitrate Membranes (Amersham, USA) using a Transblot system (Bio-Rad, USA). Immunoblotting was performed as previously described [36] using appropriate primary antibodies(anti-Cbfa1 at 1∶500 dilution, Abcam, Hong Kong) and secondary antibodies (goat anti-mouse-HRP-conjugated at 1∶5000 dilution; Abcam). Blots were further stained with DAB reagents (Maixin, FuJian China) for visualization.

## Results

### Morphology and GFP expression of antler stem cell-derived nodules

**Figure 1:**
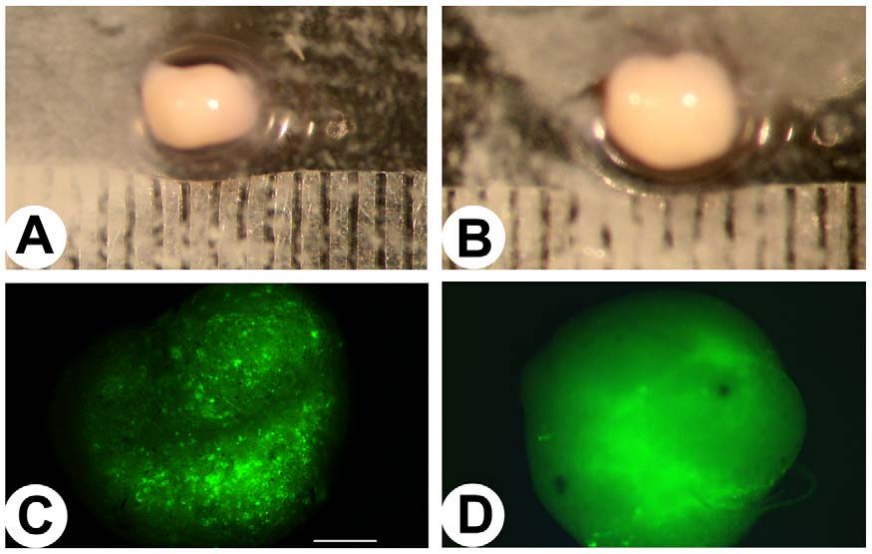
Morphology and GFP expression of antler stem cell-derived nodules. A and B: Nodule morphology. A, Nodule from the S6-infected group. B, Nodule from the control (scrambled sequence) group. Note that there was essentially no difference in shape and size between theS6-infected and the control groups. C and D: GFP expression (50x). C, Nodule from the S6-infected group. D, Nodule from the control group. Note that numerous fluorescent dots could be observed from the S6-infected nodule, but the control nodule only showed faint auto-fluorescence.

Antler stem cells began to form observable nodules 2–3 days after the initial seeding for micromass culture. The nodules increased in size as a function of culture time. By the time of harvesting (3–4 weeks after seeding), a round or oval opaque body measuring 3–4 mm in diameter was formed in each well. There was no obvious difference in size or shape detected between the nodules formed from the lentiviral-infected (Fig. 1A) and uninfected (Fig. 1B) antler stem cells.

Under the fluorescent microscope, numerous sharp bright fluorescent dots (detectable on day 7 onwards), with the shape of cultured antler stem cells, were densely populated at a given focusing plane in each nodule formed by the infected antler stem cells (Fig. 1C); whereas, just faint auto-fluorescence was detected from the nodules formed by the uninfected antler stem cells (Fig. 1D). These results confirmed that lentiviral infection was successful.

### Effects of RNAi on Cbfa1 gene expression

**Figure 2:**
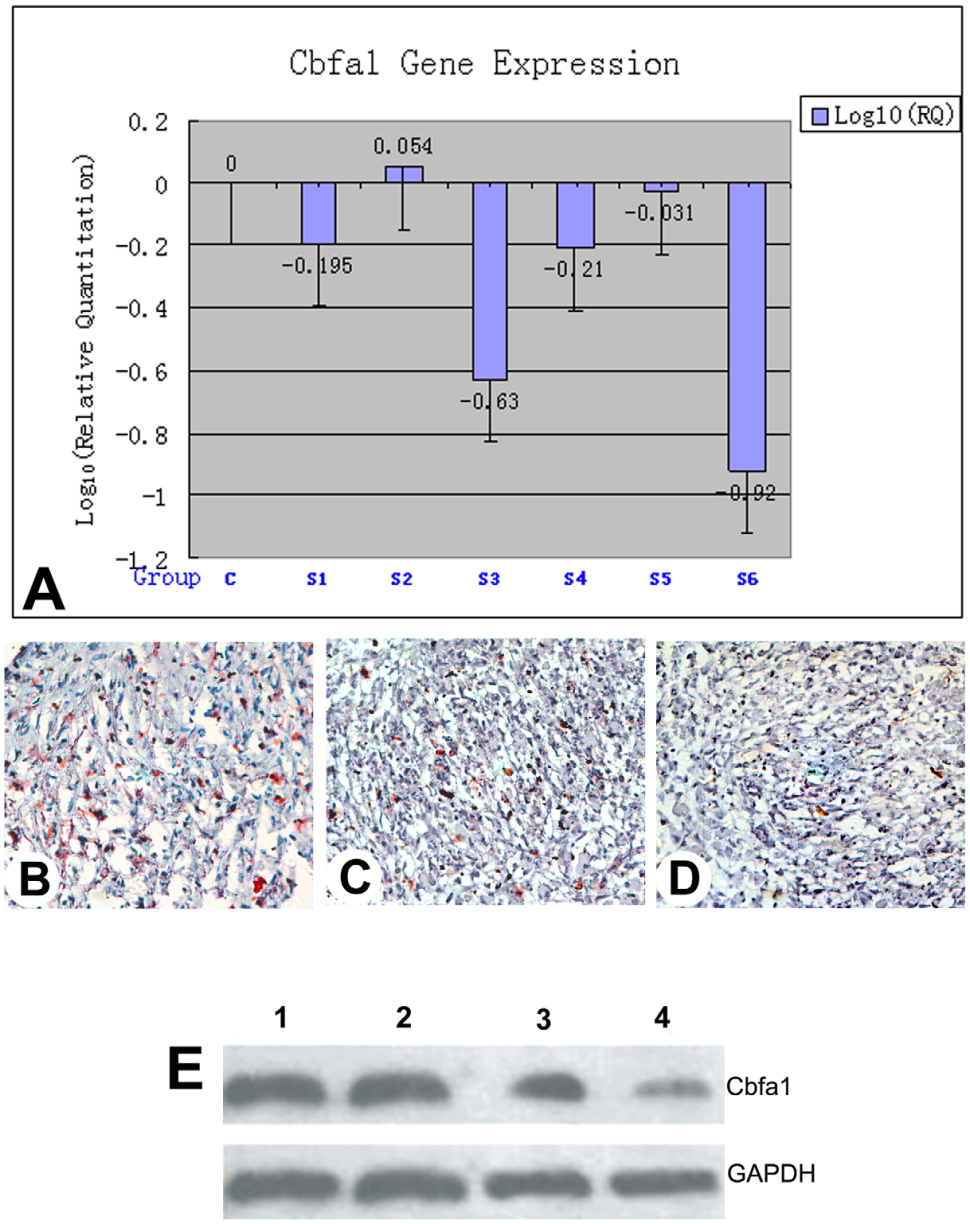
Effects of RNAi on Cbfa1 gene expression in antler stem cell-derived nodules. A: qPCRof Cbfa1. Note that five of six siRNA target sequences (S1, S3, S4, S5, S6)down-regulated Cbfa1 gene expression compared to the scrambled sequence (C), particularly the S6 sequence that had most significant effects and knocked down expression of Cbfa1 gene up to 88%. B–D: Immunohistochemistry of Cbfa1 (400X). B, Section from the uninfected nodule. C, Section from the S6-infected nodule. D, Section from the S6-infected nodule but in the absence of the primary antibody. Note that uninfected nodule tissue had the strongest staining, and the staining of the S6-infected nodule tissue was significantly weaker, but still stronger than the S6-infected nodule in the absence of the primary antibody. E: Western blot analysis of Cbfa1 gene expression. 1 and 2, Cbfa1 protein bands from the two uninfected nodules; 3 and 4, from two of the S6-infected nodules. Note that the band sizes from the uninfected nodules were substantially bigger than those from the S6-infected nodules; whereas, no difference in GAPDH (control) could be visualised amongst the bands between the infected and uninfected nodules.

qPCR results demonstrated that five out of six of our siRNA target sequences (S1, S3, S4, S5, and S6) down-regulated Cbfa1 gene expression in antler stem cell-derived nodules (Fig. 2A) compared to the negative control (scrambled RNA group). Amongst those sequences, the S6 sequence had the most dramatic effects on silencing Cbfa1 gene expression (up to 88%; P<0.01).

IHC results showed that the tissue sections from the uninfected nodules were most heavily stained by anti-Cbfa1 antibody (Fig. 2B); whereas, the staining of those from the S6-infected nodules was significantly reduced (Fig. 2C) although still more than those from the controls (Fig. 2D; the sections from the same nodule, but in the absence of the primary antibody).

Western blot analysis further confirmed the reduction in Cbfa1 expression in the S6-infected nodules compared to the controls (Fig. 2E). Therefore, we conclude that we have successfully knocked down Cbfa1 gene expression in the S6-infected nodules formed from antler stem cells.

### Effects of Cbfa 1 silencing on the expression of type I collagen and osteocalcin genes

**Figure 3:**
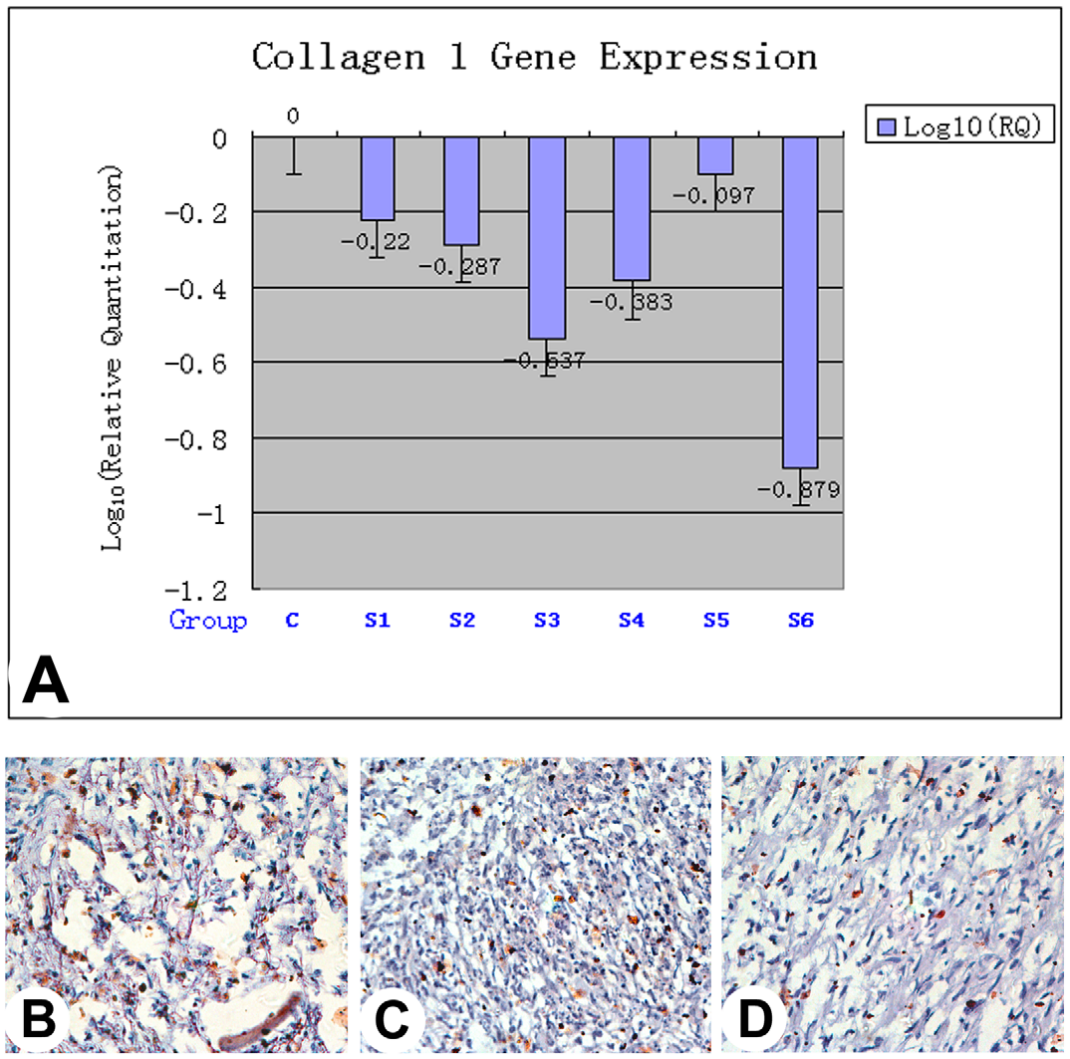
Effects of Cbfa 1 silencing on the expression of type I collagen and osteocalcin genes. A: qPCRof type I collagen. Note that all of the siRNA Cbfa1-target sequences(S1–S6) suppressed expression of downstream gene type I collagen compared to the scrambled sequence (C), particularly the S6 sequence that had most dramatic effects and knocked down expression of type I collagen gene up to 86.8%. B–D: Immunohistochemistry of osteocalcin (400X). B, Section from the uninfected nodule. C, Section from the S6-infected nodule. D, Section from the S6-infected nodule but in the absence of the primary antibody. Note that uninfected nodule had the strongest staining, and the staining of the S6-infected nodule was significantly weaker, but still stronger than the S6-infected nodule in the absence of the primary antibody.

Results from our qPCR showed that all siRNA Cbfa1-target sequences had effectively suppressed expression of downstream gene type I collagen in the micromass-cultured antler stem cells compared to the negative control (scrambled sRNA group). Consistent with RNAi knockdown of Cbfa1, the S6 sequence had the greatest suppression of type I collagen gene expression (86.8%; P<0.001; Fig. 3A).

Furthermore, IHC results demonstrated that the tissue sections from the uninfected nodules were most heavily stained by anti-osteocalcin antibody (Fig. 3B); whereas, the staining of those from the S6-infected nodules was significantly reduced (Fig. 3C) although still more than those from the controls (Fig. 3D; the sections from the same nodule, but in the absence of the primary antibody). Therefore, we conclude that the expression of both downstream genes type I collagen and osteocalcin are under the control of Cbfa1 gene in the micromass cultured antler stem cells.

### Effects of cbfa 1 silencing on chondrogenic and osteogenic differentiation

**Figure 4:**
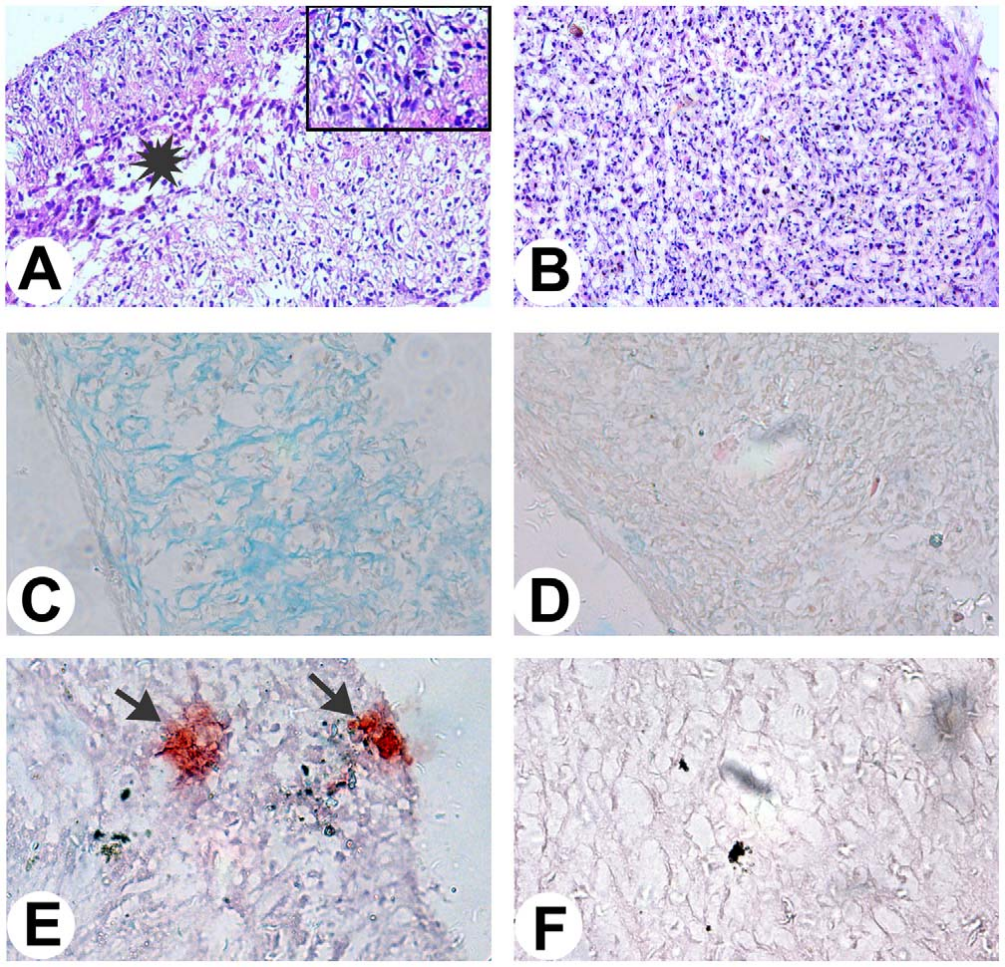
Effects of Cbfa 1 silencing on chondrogenic and osteogenic differentiation in antler stem cell-derived nodules. A and B: Histology (200X). A, From the uninfected nodule, remodelling process (asterisk), chondroclast-like cells (arrow) and typical chondrocytes residing in the lacunae (inset) could be observed in the central region of the tissue. B, From the S6-infected nodule, no sign of EO in the S6-infected nodule could be detected. C and D: Alcian blue staining (400X). C, From the uninfected nodule. B, From the S6-infected nodule. Note that the section from the uninfected nodule was heavily stained, whereas from the S6-infected nodule was barely stained. E and F: Alizarin red staining (400X). E, From the uninfected nodule. F, From the S6-infected nodule. Note that two foci on the tissue section from the uninfected nodule were heavily stained (arrows), whereas the tissue section from the S6-infected nodule was not stained.

Histological examination demonstrated that in the lentiviral infected nodules the seemingly undifferentiated antler stem cells (small and homogenous) were evenly distributed (Fig. 4A). In contrast, in the uninfected nodules, cell remodelling process had taken place in the central region of each nodule; chondroclasts/osteoclast-like cells were frequently encountered in the remodelling zone (Fig. 4B). Some cells in the vicinity of the remodelling zone were hypertrophied and resided in the typical lacunae (Fig. 4A inset) surrounded by abundant extracellular matrix (ECM), resembling chondrocytes that are found in cartilage in vivo. The ECM from the uninfected nodules was heavily stained with alcian blue (Fig. 4C), whereas that from the infected nodules was essentially negative to the staining (Fig. 4D), suggesting that the infected nodules does not contain sulphated proteoglycans, an essential component of cartilage matrix. In agreement with the results of alcian blue staining, alizarin red stained the tissue sections from the uninfected nodules with distinctive pattern (Fig. 4E), but did not stain those from the infected nodules (Fig. 4F). These results indicate that both chondrogenesis and osteogenesis had taken place in the antler stem cell formed nodules in the control uninfected group, and RNAi had effectively impeded the progression of EO processes.

## Discussion

The inability to repair defects of articular cartilage (AC) is mainly attributed to its avascular nature, as AC repair requires specific cells being brought in by blood vessels [2]. Uniquely, antler cartilage is richly infiltrated with blood vessel networks, hence is capable of effective growth, repair and regeneration [12], [20]. Interestingly, antler stem cells can also form avascular cartilage just by changing the environment in which these cells reside [23], [37]. Understanding the molecular mechanism of this phenotypic switch could open a new avenue for effective AC repair, provided that antler chondrogenesis is also regulated by the same signalling pathways. The present study convincingly demonstrated that effective knockdown of the key molecule Cbfa 1 in antler stem cells during endochondral ossification (EO) significantly impaired expression of the down-stream osteogenic genes including osteocalcin and type I collagen (Fig. 3), and hindered the progresses of chondrogenesis and osteogenesis in vitro (Fig. 4). Therefore, the antler cartilage model is uniquely suited for the identification of novel molecules for the phenotype switch between vascular and avascular states. These putative molecules, if identified, would be invaluable for the development of effective means for intrinsic AC repair.

### Validity of the approach taken in the present study

The conclusion, antler chondrogenesis is regulated by the same signalling pathway that prevails in the somatic counterpart, drawn from this study is all based on the in vitro models. We acknowledge that this in vitro approach may not fully represent the biology in vivo. However, investigation into chondrogenesis is not always practical to carry out using live animals; hence several in vitro culture systems have been developed in the field to reproduce the events that occur during the process in vivo. Amongst these systems, micromass culture has been found to be superior for the expression of full phenotypic repertoire of cartilage including cellular morphology, matrix characteristics, and the apoptotic program [33], [38]. In the present study, we successfully adopted the system using antler stem cells and generated 2–4 mm in diameter solid cell nodules. Histological examination revealed that typical EO had taken place in these nodules from the uninfected control group, which allowed us to effectively evaluate the influence of RNAi to Cbfa1 gene on chondrogenesis of antler stem cells in the infected group.

The RNAi technique used in the present study has been widely adopted by researchers in recent years to silence the expression of many target genes in mammalian cells because of their high specificity and apparent non-toxicity [39]. In addition, in our study we chose a lentiviral vector as our siRNA delivery vehicle because they can infect both dividing and nondividing cells at a high efficiency and achieve long-term stable RNAi-mediated knockdown [40], [41]. Our lentiviral vector encodes GFP as a marker for infectivity, which had allowed us to track the RNAi knockdown cells in real-time. Therefore, we believe the in vitro approach taken in our present study is valid and effective for achieving our aims.

The reason that the siRNAS2 failed to inhibit expression of Cbfa1 gene but effectively interfered with expression of type I collagen gene is subject to speculation. Through the alignment we know that S2 sequence binds to the 3′untranslated region (UTR) of the Cbfa1 gene, and according to the report that the 3′ UTR region of a geneis rich in regulatory protein binding sites and these regulatory proteins may interfere with the binding of the complex of siRNA and endonuclease with mRNA [42], hence affect the effects of siRNA on expression of the target gene. Therefore, this is probably the reason why S2 did not show the inhibitory effects on Cbfa1 gene expression. For an unaccountable reason, osteogenesis of the S2 group nodule was impeded and hence expression of type I collagen gene was affected, although Cbfa1 gene expression seemed normal. Hence, there may have thus far unknown alternative mechanisms involved in down-regulation of Col I, which is not through reduction of Cbfa1. The 3′ UTR region of a gene should be avoided in the future siRNA sequence design in order not to cause downstream complication.

### Histogenesis of vascularised cartilage

Unlike somatic EO where vasculature is established through invasion of blood vessel into the calcified cartilage [43], the process of antler cartilage formation is unique in that the angiogenesis is an integral part of it [44]. Histological makeup of the growth centre in a developing antler is comprised of four zones: mesenchyme, precartilage, transition and cartilage distoproximally [14], [45]. Cells in the mesenchymal zone are the least and in the cartilage zone are the most differentiated. In the mesenchymal zone cells are distributed more or less evenly, whereas in the precartilage zone prechondroblasts are separated by the newly forming cell aggregates with the longitudinally elongated shape (Fig. S2A). These elongated cell aggregates gradually extend in length towards more proximal antler zones, and eventually become continuous channels when reaching the cartilage zone (Fig. S2B). Blood vessel wall markers, like smooth muscle actin [44] and CD146 [46], were found to be expressed by cells of these aggregates, discontinuous and continuous channels, indicating these cells belong to endothelial and supporting cells (Fig. S1A and S1B). Further research revealed that the majority of cells in the lower mesenchymal zone are labelled with BrdU (Fig. S2C); in contrast, proliferating cells in the precartilage zone are solely confined to these newly differentiated endothelial and supporting cells (Fig. S2D), suggesting that the centres of antler chondrogenesis and angiogenesis are distinct and spatially separate in the growing antlers: the former is located in the mesenchymal zone and the latter in the precartilage zone [44], [45].

While it may be convincing that angiogenesis initiates in the zone of precartilage, the origin of the angiogenic cells has never been determined. There is reason to believe that these angiogenic cells are, like prechondroblasts, also differentiated from the mesenchymal cells. Our working hypothesis is that during evolution deer adopted antlers as their secondary sexual appendages. In order to be used in the rutting season, deer must complete the growth of this large bony organ (about a metre in length) within a very limited time (approximately 60 days). This means that antlers must elongate at a rate of over 1 cm a day. As the fastest way to form bone tissue, EO (cartilage is laid down first then replaced by bone through remodelling) has to be the choice; however cartilage tissue has its own drawback. Due to its avascular nature, cartilage can only obtain nourishment through diffusion from the surroundings, but the diffusion distance is limited [47]. To complete antler growth in time, antler mesenchymal cells must maintain an extremely fast rate of proliferation and sequential differentiation into cartilage, but this requires the progression of tissue remodelling and bone replacement to keep the same pace in order to allow the cells in the deepest cartilage region reaching the source of nutrients and oxygen. However, this is not the case in antler as an extensive cartilage mass was remained intact in the growth centre, so the remodelling must have lagged behind. Instead of initiating a secondary ossification centre in reaction to the hypoxia condition in the heart of the extensive cartilage mass, like those that prevail in long bone formation, deer invented a way to allow antler mesenchymal cells in the area where subjects to hypoxia to differentiate into endothelial cells, which are then to form vascular system. In so doing, not only is the fast rate of cartilage formation effectively maintained during antler formation, but also the slower progression of cartilage remodelling and bone replacement will not become a limiting factor to hinder rapid antler growth. If this analysis withstands testing, it is probably no surprise why the classic cell signalling pathways that are identified in the avascular somatic cartilage also prevail in the vascularised antler cartilage.

### Extrinsic factors and the formation of vascularised cartilage

The fact that the same antler stem cells can form vascular or avascular cartilage just depending on the milieu they reside highlights the importance of extrinsic factors to this phenotype switch. Deciphering this milieu is obviously the key should the antler cartilage model be translated to AC repair. Based on our previous studies, we believe both endocrine (androgen hormones [48], [49] and IGFs [50]) and paracrine factors (deer skin derivatives, [14]) contribute to the formation of this unique milieu. When taken away from the deer paracrine factors, antler stem cells can only form bone tissue with a narrow band of avascularised cartilage (Fig. S3A), reminiscent of long bone growth plate cartilage [23]. Nevertheless, in the absence of the deer endocrine factorsa secondary ossification centre, rather than vascular system, would initiate in the deep part of the antler stem cell-formed cartilage mass (Fig. S3B) [37], just like what happens in the long bone formation. Full identification of these deer endocrine and the paracrine factors would greatly facilitate the progress towards reconstitution of the unique milieu for triggering effective intrinsic repair of AC defects.

Furthermore, antler cartilage has a unique expression pattern for Col X. Instead of being localized solely to the lower hypertrophic zone of a long bone growth plate [51], [52], distribution of Col X in antler cartilage is far more extensive right from the precartilage zone through transition zone to the mineralized region of the cartilage zone [53]. Iyama et al. [54] proposed a role of Col X in vascular invasion and Price et al [53] thought that this may have explained why antler cartilage has the extensive network of vascular channels. It is known that Col X expression is also under the control by Cbfa1 [55], [56] and successful silencing of Cbfa1 gene expression may have also suppressed Col X expression in this study, which would have played important role in the impediment of EO of antler stem cells in our micromass culture.

Overall, the present study demonstrated that silencing of Cbfa 1 gene expression in antler stem cells during endochondral ossification (EO) effectively impaired expression of osteocalcin and type I collagen, the down-stream osteogenic genes; and hindered the progresses of EO. Identification of novel molecules for the cartilage phenotype switch between vascular and avascular states through investigating antler cartilage model would be invaluable for the development of effective means for intrinsic AC repair.

## Supporting Information

Figure S1 **Lentiviral vector system.** A, pLVTHM; B. pCMV; C, pMD2. G.
(TIF)

Figure S2 **Centres of antler chondrogenesis and angiogenesis.** A: Tissue section from the antler precartilage zone. Note that the cells were separated by the newly forming cell aggregates (arrow), which were stained with smooth muscle actin (blood vessel wall marker) antibody. B: Section from the antler cartilage zone. Note that the elongated cell aggregates had become continuous channels and the wall of these channels were stained with antibody of smooth muscle actin (arrow). C: Section from the antler mesenchymal zone. Note that majority of the cells in the lower zone were labelled with BrdU (marker for mitosis). D: Section from the antler precartilage zone. Note that proliferating cells were solely confined to these newly forming cell aggregates (arrow; smooth muscle actin positive cells).
(TIF)

Figure S3 **Chondrogenesis of antler stem cells.** A: Tissue section of the antlerogenic tissue cultivated in a diffusion chamber in vivo. Note that antler stem cells formed a narrow band of avascularised cartilage (asterisk) above a layer of trabecular bone (star). B: Tissue section of the antlerogenic tissue that was co-transplanted with deer skin onto a nude mouse. Note that a secondary ossification centre (arrow) was initiated in the heart of the extensive cartilage mass.
(TIF)
