# Supplementary figures and images for: Lentiviral-Mediated RNAi Knockdown of Cbfa1 Gene Inhibits Endochondral Ossification of Antler Stem Cells in Micromass Culture
Source: PLoS One. 2012 Oct 9;7(10):e47367. doi: 10.1371/journal.pone.0047367 (PMC3467256; doi:10.1371/journal.pone.0047367)

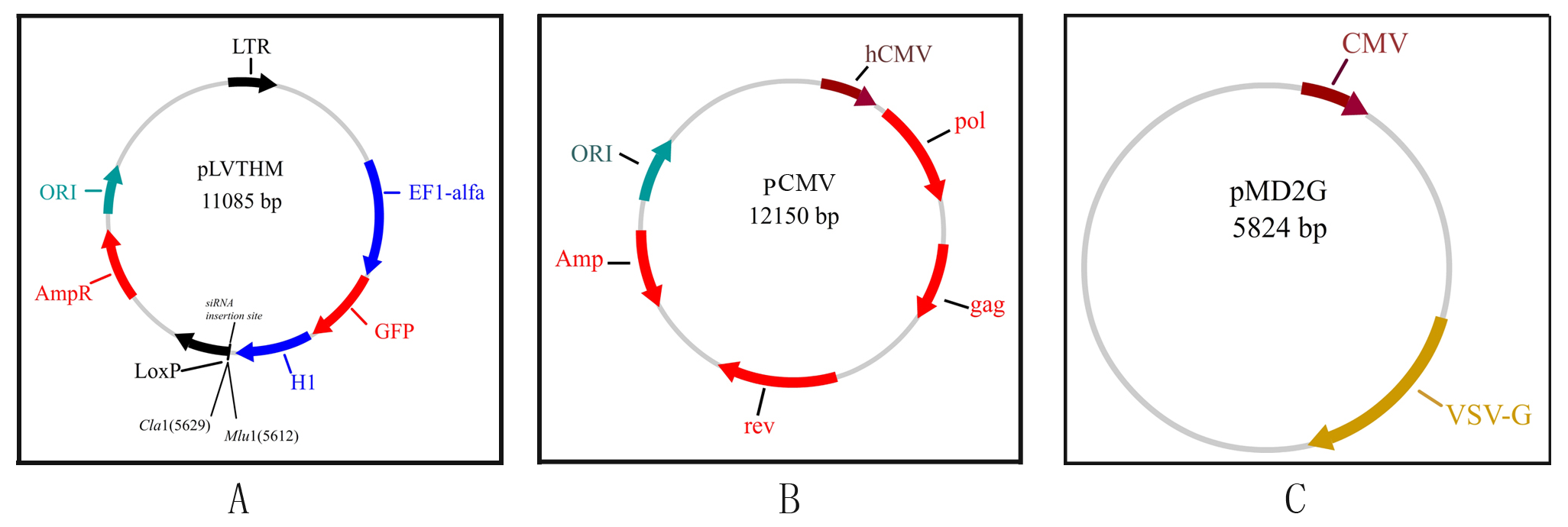

Supplement: Figure S1 — Lentiviral vector system. A, pLVTHM; B. pCMV; C, pMD2. G. (TIF) [file pone.0047367.s001.tif]

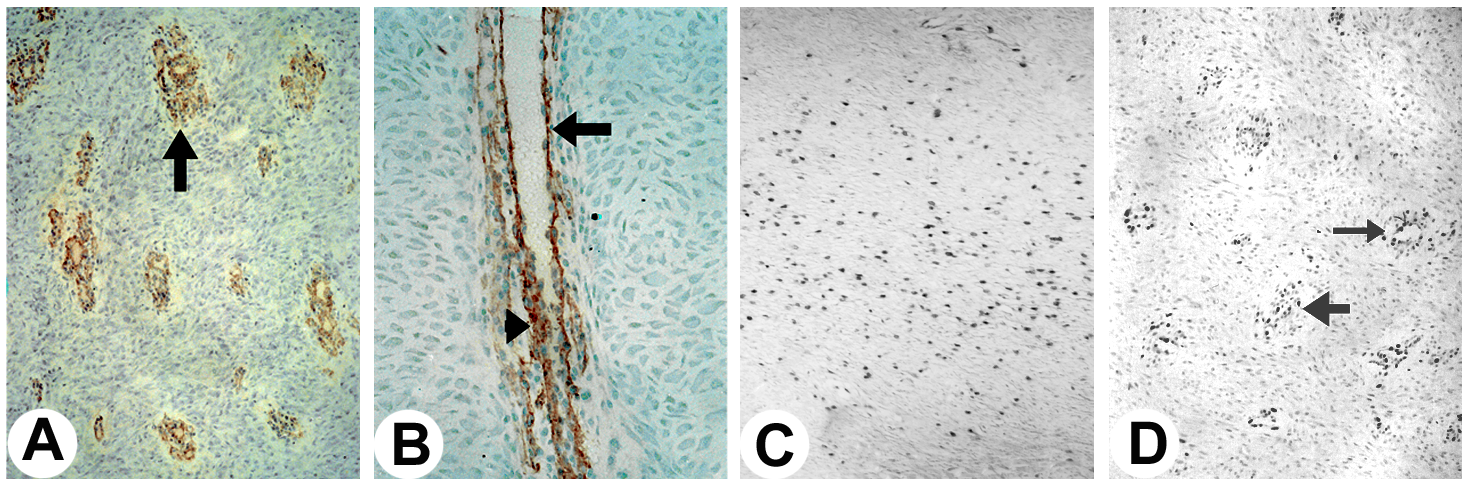

Supplement: Figure S2 — Centres of antler chondrogenesis and angiogenesis. A: Tissue section from the antler precartilage zone. Note that the cells were separated by the newly forming cell aggregates (arrow), which were stained with smooth muscle actin (blood vessel wall marker) antibody. B: Section from the antler cartilage zone. Note that the elongated cell aggregates had become continuous channels and the wall of these channels were stained with antibody of smooth muscle actin (arrow). C: Section from the antler mesenchymal zone. Note that majority of the cells in the lower zone were labelled with BrdU (marker for mitosis). D: Section from the antler precartilage zone. Note that proliferating cells were solely confined to these newly forming cell aggregates (arrow; smooth muscle actin positive cells). (TIF) [file pone.0047367.s002.tif]

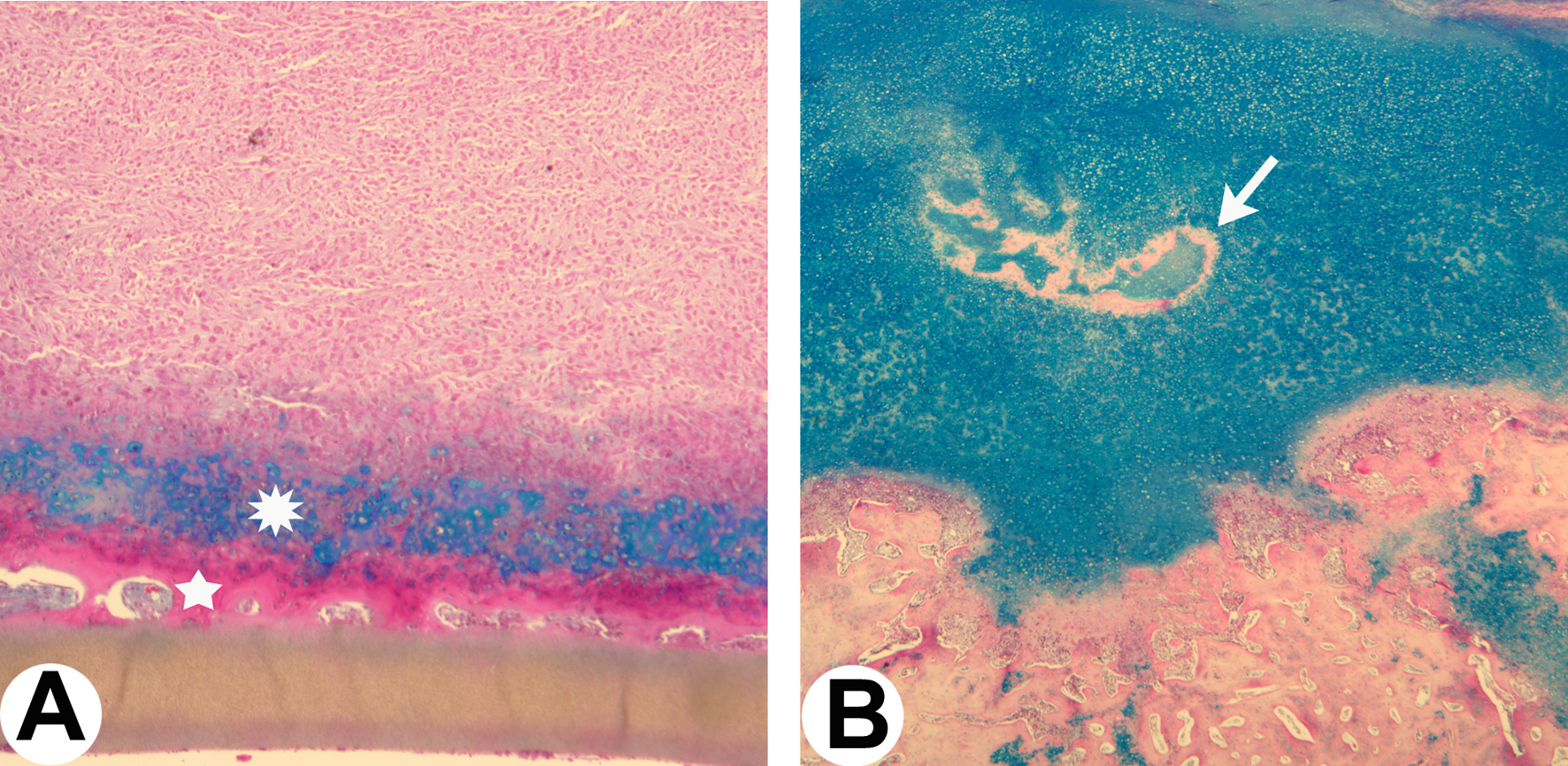

Supplement: Figure S3 — Chondrogenesis of antler stem cells. A: Tissue section of the antlerogenic tissue cultivated in a diffusion chamber in vivo. Note that antler stem cells formed a narrow band of avascularised cartilage (asterisk) above a layer of trabecular bone (star). B: Tissue section of the antlerogenic tissue that was co-transplanted with deer skin onto a nude mouse. Note that a secondary ossification centre (arrow) was initiated in the heart of the extensive cartilage mass. (TIF) [file pone.0047367.s003.tif]
